# Composition and diversity of gut microbiota in non-erosive reflux disease

**DOI:** 10.3389/fmicb.2026.1795756

**Published:** 2026-04-22

**Authors:** Xiuxiu Wei, Gaoxiang Wang, Yuchen Wei, Tai Zhang, Mengxiong Lu, Luzhou Xu, Beihua Zhang, Xudong Tang

**Affiliations:** 1 Department of Gastroenterology, Affiliated Hospital of Nanjing University of Chinese Medicine, Nanjing, China; 2 Department of Endocrinology, Affiliated Hospital of Nanjing University of Chinese Medicine, Nanjing, China; 3 Institute of Digestive Diseases, Xiyuan Hospital of China Academy of Chinese Medical Sciences, Beijing, China; 4 Graduate College, Beijing University of Chinese Medicine, Beijing, China; 5 Peking University Traditional Chinese Medicine Clinical Medical School (Xiyuan), Peking University Health Science Center, Beijing, China

**Keywords:** 16S rRNA sequencing, gut microbiota, human, microbial biomarker, non-erosive reflux disease

## Abstract

**Objective:**

Non-erosive reflux disease (NERD) is a prevalent gastrointestinal disorder with complex pathophysiology. Recent evidence suggests a potential role of gut microbiota in its development. This study aimed to characterize the gut microbiota in patients with NERD, and to explore microbial biomarkers for disease differentiation.

**Methods:**

We enrolled 40 patients with NERD, along with 18 healthy controls (HCs). Fecal samples were collected and analyzed using 16S ribosomal RNA (rRNA) gene sequencing. Gut microbial diversity and composition, linear discriminant analysis effect size (LEfSe), and receiver operating characteristic (ROC) curves were evaluated.

**Results:**

The microbial structure and composition of NERD patients were distinct from those of HCs. Alpha diversity was significantly lower in NERD patients than in controls (*p* < 0.01). At the phylum level, Actinobacteriota was increased, while Bacteroidota and Proteobacteria were decreased in NERD patients. At the genus level, *Faecalibacterium* and *Bacteroides* were decreased, whereas *Streptococcus*, *Blautia*, *Bifidobacterium*, and *Enterococcus* were enriched in NERD patients. Additionally, LEfSe was developed to identify several bacterial genera that can differentiate patients with NERD from those HCs. Furthermore, the area under the curve (AUC) value of *Streptococcus* for distinguishing NERD from HCs was 0.9333, indicating exceptionally high diagnostic power.

**Conclusion:**

This study identified microbiota dysbiosis of gut microbiota in NERD patients. *Streptococcus* showed extremely high diagnostic efficacy, which can be used as a microbial biomarker, and may serve as potential therapeutic target for NERD.

## Introduction

The gut microbiota is a complex ecosystem composed of a vast and diverse array of microorganisms, including bacteria, fungi, archaea, and viruses, that colonize the intestinal tract and form a symbiotic relationship with the host. It is influenced by various factors such as genetics, age, geography, diet, exercise, and emotional state, research has confirmed that gut microbiota is closely associated with diseases (Schmidt et al., 2018). The complex microorganisms in the human gastrointestinal tract are becoming key participants in influencing human health and diseases (Heintz-Buschart and Wilmes, 2018). Substantial evidence indicates that alterations in gut microbiota serve as a critical factor in the development and progression of numerous local and systemic diseases (Gebrayel et al., 2022), which coincides with the holistic concept of traditional Chinese medicine. With the implementation of the Human Microbiome Project and the development of human gut metagenomics, interest in gut microbiota has grown exponentially, making it a research hotspot across multiple disciplines. Furthermore, the maturation and advancement of 16S ribosomal RNA (rRNA) sequencing technology have provided technical support for in-depth investigations of gut microbiota. The changes in gut microbiota and metabolites have attracted widespread attention in the pathogenesis of gastrointestinal motility disorders. It plays a crucial role in the occurrence and development of the disease (Quigley and Spiller, 2016).

Gastroesophageal reflux disease (GERD) is a chronic gastrointestinal motility disorder characterized by the retrograde flow of gastroduodenal contents into the esophagus, leading to typical symptoms of heartburn and regurgitation. It imposes a substantial global health burden, with a reported prevalence of 15–20% worldwide (Chapelle et al., 2021). Based on endoscopic findings, GERD is categorized into three primary subtypes: non-erosive reflux disease (NERD), reflux esophagitis (RE), and Barrett’s esophagus (BE), among which over 70% are NERD (Eusebi et al., 2018; Maret-Ouda et al., 2020). With improvements in living standards and changes in behavioral patterns and dietary habits, the prevalence of GERD has shown an increasing trend (Chinese Medical Association and the Chinese Society of Gastroenterology, 2019; Katzka and Kahrilas, 2020). A survey indicated that in 2019, over 780 million people worldwide suffered from GERD, representing 77.53% increase compared to 1990 (Zhang et al., 2022). GERD is characterized by complex symptoms and features chronic recurrent episodes, which severely impair patients’ quality of life and result in substantial consumption of healthcare and social resources (Chatila et al., 2020; Eusebi et al., 2018; Maret-Ouda et al., 2020). It has become a globally recognized health concern.

The underlying pathogenesis of NERD is multifactorial. Dysfunction of the lower esophageal sphincter and gastrointestinal motility disorders leading to displacement of gastric contents are considered the primary pathogenic mechanisms (Lin et al., 2019). Currently, first-line pharmacotherapy primarily relies on acid-suppressive agents, particularly proton pump inhibitors (PPIs). However, a significant proportion of patients experience incomplete symptom relief or rapid recurrence following discontinuation, highlighting the need for a deeper understanding of novel therapeutic targets (Fass and Sifrim, 2009; Gyawali et al., 2018). Abnormal esophageal acid exposure in GERD patients is not due to excessive gastric acid secretion. Therefore, improving gastrointestinal function rather than simply inhibiting acid secretion is the fundamental approach to preventing and treating GERD. Previous studies had confirmed that modulating the gut microbiota can promote gastrointestinal motility and positively alleviate symptoms of gastrointestinal motility disorders (Bai et al., 2022; Jiang et al., 2020; Tian et al., 2020). More and more studies had gradually revealed the objective phenomenon of gut microbiota disorder in NERD, and gut microbiota is expected to become a potential therapeutic target for NERD.

In this study, we collected fecal samples from the NERD patients and healthy controls (HCs) and delineated the community structure of the gut microbiome of NERD patients based on 16S rRNA gene sequencing. In addition, laying the foundation for exploring potential therapeutic targets and mechanisms of NERD.

## Materials and methods

### Protocol approvals and participant consent

This work was supported by the National Key R&D Program of China (Grant No. 2019YFC1709604). This clinical study was approved by the Ethics Committee of the Xiyuan Hospital of China Academy of Chinese Medical Sciences (Ethics Approve No. 2020XLA008-3) and registered at ClinicalTrials.gov (Identifier: NCT04340297). The study protocol adhered to the principles outlined in the Declaration of Helsinki. All participants voluntarily provided informed consent.

### Subject recruitment

A total of 58 participants were enrolled, including 40 patients diagnosed with NERD and 18 HCs. The diagnosis of NERD was established according to the 2020 Chinese Expert Consensus on Gastroesophageal Reflux Disease (Chinese Society of Gastroenterology and Chinese Medical Association, 2020). Inclusion criteria for NERD patients were: (1) age between 18 and 70 years; (2) typical symptoms of reflux and heartburn with negative impact on quality of life; (3) endoscopic examination within the past year confirmed absence of BE or esophageal mucosal breaks; (4) showing a positive response to an empirical PPIs trial. HCs were age- and sex-matched individuals with no history of gastrointestinal diseases, metabolic disorders, or psychiatric conditions.

Exclusion criteria for subjects were as follows: (1) use of microecological preparations or antibiotics within the previous 2 weeks; (2) had active peptic ulcer, gastrointestinal hemorrhage, severe dysplasia of gastric mucosa or suspected malignant change, achalasia or postoperative achalasia; (3) had organic diseases of the digestive system (such as acute and chronic pancreatitis, cirrhosis, etc.), or systemic diseases that affect the gastrointestinal motility, such as hyperthyroidism, diabetes mellitus over 10 years, chronic renal insufficiency, spirit (the score of self-rating anxiety scale and self-rating depression scale shows severe anxiety or depression), nervous system diseases, etc.; (4) severe organ diseases such as heart, liver and kidney (such as alanine aminotransferase, aspartate transaminase more than 2 times of normal value), hematopoietic system diseases and tumors; (5) pregnancy or breastfeeding; (6) with a history of nervous system disease and mental disease.

### Sample collection and processing

Fecal samples were collected approximately 3–5 g from each participant using sterile containers and immediately stored on ice. Within 2 h of collection, samples were flash-frozen in liquid nitrogen and subsequently stored at −80 °C until DNA extraction. Female participants were advised to avoid providing samples during menstruation.

### DNA extraction and PCR amplification

Total microbial DNA was extracted from approximately 200 mg of each fecal sample using the E.Z.N.A.® soil DNA Kit (Omega Bio-tek, Norcross, GA, U.S.) following the manufacturer’s instructions. DNA quality and concentration were assessed by 1% agarose gel electrophoresis and a NanoDrop 2000 spectrophotometer (Thermo Scientific, USA). The V3-V4 hypervariable region of the bacterial 16S rRNA gene were amplified with primer pairs 338F (5′-ACTCCTACGGGAGGCAGCAG-3′) and 806R (5′-GGACTACHVGGGTWTCTAAT-3′) using an ABI GeneAmp® 9,700 PCR thermocycler (ABI, CA, USA). Each 20 μL PCR reaction contained 4 μL of 5× FastPfu buffer, 2 μL of 2.5 mM dNTPs, 0.8 μL of each primer (5 μM), 0.4 μL of FastPfu DNA polymerase, and 10 ng of template DNA. Amplification conditions were: 95 °C for 3 min; 27 cycles of 95 °C for 30 s, 55 °C for 30 s, and 72 °C for 45 s; and a final extension at 72 °C for 10 min. All reactions were performed in triplicate. PCR products were purified using the AxyPrep DNA Gel Extraction Kit (Axygen Biosciences, Union City, CA, USA) and quantified with a Quantus™ Fluorometer (Promega, USA).

### Illumina MiSeq sequencing

Purified amplicons were pooled in equimolar and paired-end sequenced on an Illumina MiSeq PE300 platform (Illumina, San Diego, USA) following standard protocols at Majorbio Bio-Pharm Technology Co. Ltd. (Shanghai, China).

### Processing of sequencing data

Raw 16S rRNA gene sequencing reads were demultiplexed and quality-filtered using fastp version 0.19.6^1^ and merged by Flash version 1.2.11^2^ with the following criteria: (1) the 300 bp reads were truncated at any site receiving an average quality score of <20 over a 50 bp sliding window, and the truncated reads shorter than 50 bp were discarded, reads containing ambiguous characters were also discarded; (2) Paired reads were merged with a minimum overlap of 10 bp and a maximum mismatch ratio of 0.2; (3) Operational taxonomic units (OTUs) were clustered at 97% similarity using Uparse version 11,^3^ and chimeric sequences were identified and removed. Taxonomic classification was performed using the RDP classifier version 2.13^4^ against the 16S rRNA database (v138) using confidence threshold of 0.7. All samples were rarefied to the minimum sequencing depth for downstream analyses.

### Statistical analysis

(1) OTU Analysis: The UPARSE software (see text footnote 3, version 11) was employed to perform OTU clustering of sequences based on a 97% similarity threshold, with concurrent removal of chimeric sequences. (2) Taxonomic annotation: The RDP Classifier (see text footnote 4, v. 2.13) was utilized to assign taxonomic annotations to each individual sequence. Sequences were aligned against the Silva 16S rRNA database (v138) with an alignment threshold set at 70%, followed by rarefaction of sequences to the minimum sample sequence count across all samples. (3) Rarefaction curve analysis: Rarefaction curves were generated at the OTU level (97% sequence similarity) using the mothur software (https://www.mothur.org/wiki/Download_mothur, v. 1.30.2) to compute the Sobs and Shannon indices under repeated random subsampling. Curves were visualized using R (v. 3.3.1). (4) Alpha diversity analysis: Alpha diversity indices were computed using mothur software (https://www.mothur.org/wiki/Download_mothur, v. 1.30.2). For testing differences in alpha diversity indices among groups, the Wilcoxon rank-sum test was applied for pairwise comparisons between two groups, while the Kruskal–Wallis rank-sum test was used for comparisons involving more than two groups. The false discovery rate (FDR) method was implemented to correct for multiple testing. (5) Beta diversity analysis: Principal coordinate analysis (PCoA) was conducted based on weighted UniFrac distances. Permutational multivariate analysis of variance (Adonis) was used to evaluate group differences. (6) Species composition analysis: For each group, the species abundance values across samples were averaged. Microbial communities with a relative abundance of less than 0.01% were aggregated into a category labeled “Others” for subsequent analysis. Data tables retrieved from the “tax_summary_a” folder were used. (7) Linear discriminant analysis effect size (LEfSe) multilevel species differential discriminant analysis^5^: Differential tests were carried out across multiple taxonomic levels (phylum, class, order, family, genus, and species). This analysis aimed to identify microbial species that exerted a significant differential impact on the classification and distinction of samples. Analyses and visualizations were performed in R (v. 3.3.1).

## Results

### Sequencing quality assessment

**Figure 1:**
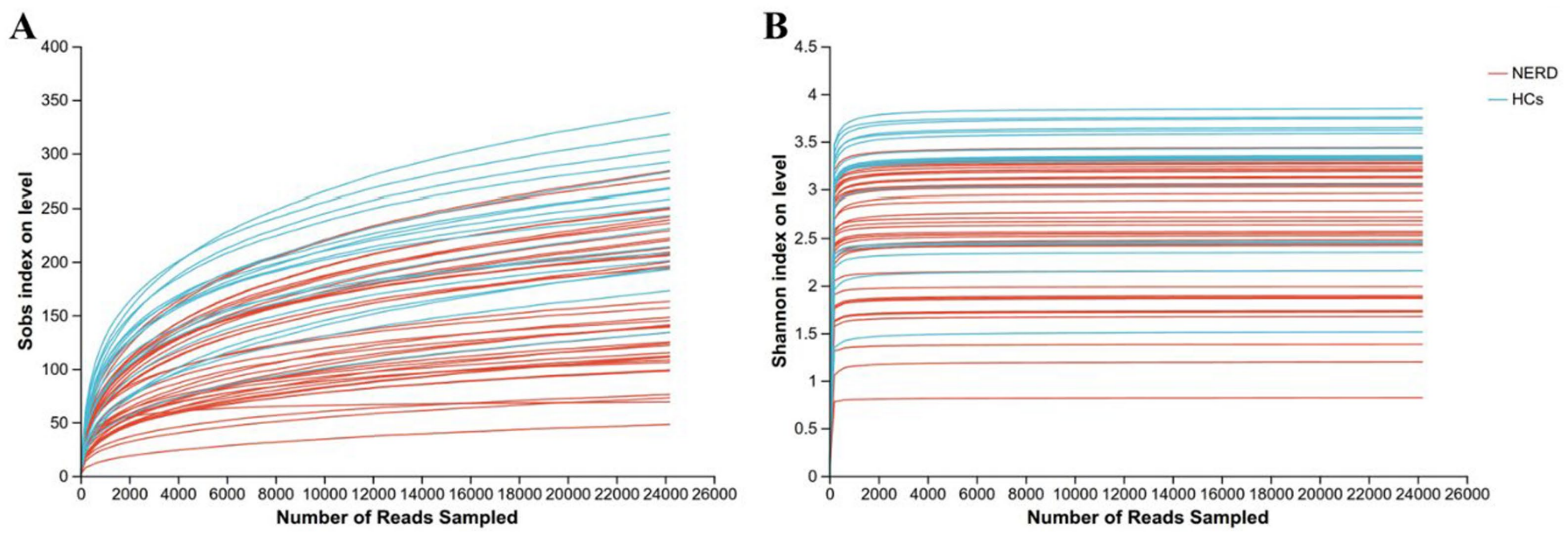
Rarefaction curves of gut microbiota in 58 participants: **(A)** Sobs rarefaction curve and **(B)** Shannon rarefaction curve.

After quality filtering and chimera removal, a total of 1,404,006 high-quality sequences were obtained from 58 fecal samples. Rarefaction curves for the Sobs and Shannon indices reached plateaus, indicating that the sequencing depth was sufficient to capture the majority of microbial diversity (Figure 1). Each sample was rarefied to 24,207 valid sequences, with a total of 20 phyla, 31 classes, 73 orders, 132 families, 341 genera, 674 species, and 1,064 OTUs identified for subsequent analyses.

### NERD significantly altered the gut microbiota diversity

#### Diversity of intestinal microbial communities

**Figure 2:**
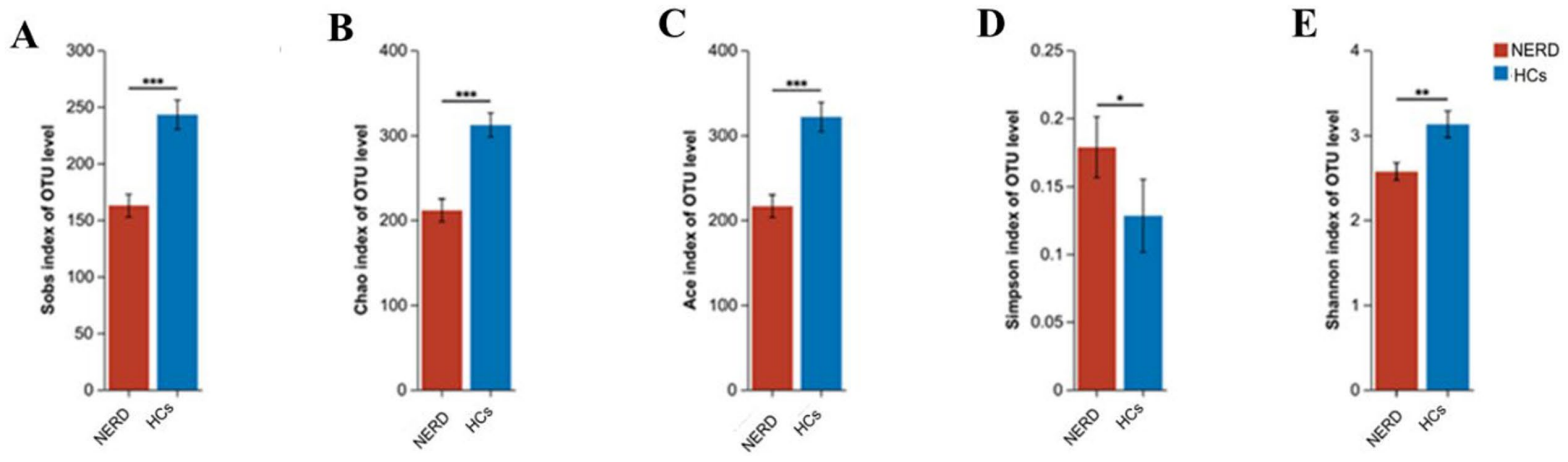
Comparison of alpha diversity indices between NERD and HC groups. **(A)** Sobs index; **(B)** Chao index; **(C)** Ace index; **(D)** Simpson index; **(E)** Shannon index. *p < 0.05, **p < 0.01, ***p < 0.001.

Alpha diversity indices (Sobs, Chao, Ace, Shannon) were significantly lower in the NERD group compared with HCs (*p* < 0.01), while the Simpson index was significantly higher (*p* < 0.05) (Figure 2).

**Figure 3:**
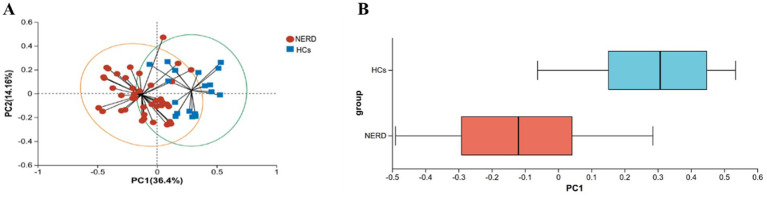
Beta diversity analysis of gut microbiota between NERD and HC groups. **(A)** Principal coordinate analysis (PCoA) plot based on weighted UniFrac distances at the OTU level. Each point represents an individual sample. **(B)** Box plot showing the distribution of weighted UniFrac distances between groups.

Beta diversity analysis revealed a distinct separation between the NERD and HC groups. PCoA based on weighted UniFrac distances showed that samples from the NERD group clustered separately from those of the HC group, and the difference was statistically significant (*p* < 0.01, Figure 3). These findings indicate that NERD is associated with reduced richness and altered structure of the gut microbiota.

#### Differential bacterial composition and abundance in NERD and HC groups

**Figure 4:**
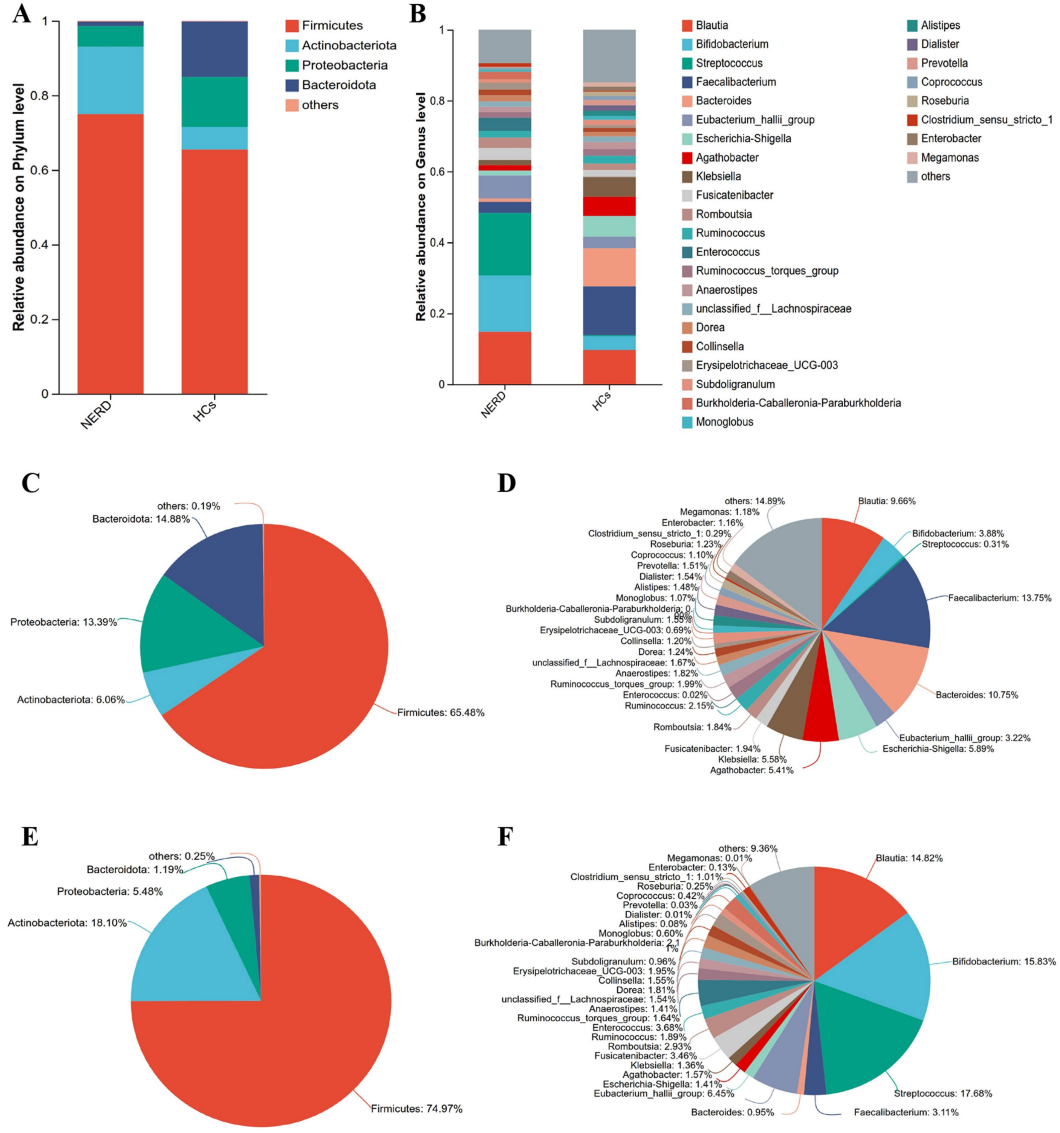
Variations in fecal microbiota composition between NERD and HC groups. **(A)** Community barplot analysis at the phylum level. **(B)** Community barplot analysis at the genus level. **(C)** Community analysis pieplot on phylum level in HCs. **(D)** Community analysis pieplot on genus level in HCs. **(E)** Community analysis pieplot on phylum level in NERD. **(F)** Community analysis pieplot on genus level in NERD.

At the phylum level, the relative abundance of *Actinobacteriota* was significantly higher in NERD patients (*p* < 0.01), whereas *Proteobacteria* and *Bacteroidota* were significantly reduced (*p* < 0.001 and *p* < 0.05, respectively; Figure 4, Table 1).

**Table 1:** Comparison of relative species abundance at the phylum and genus levels.

Fecal microbiota	HC group	NERD group	*p*-value
Firmicutes	65.48%	74.97%	>0.05
Actinobacteriota	6.06%	18.1%^**^	<0.01
Proteobacteria	13.39%	5.48%^*^	<0.05
Bacteroidota	14.88%	1.19%^***^	<0.001
*Blautia*	9.66%	14.82%^*^	<0.05
*Bifidobacterium*	3.88%	15.83%^*^	<0.05
*Faecalibacterium*	13.75%	3.11%^***^	<0.001
*Bacteroides*	10.75%	0.95%^***^	<0.001
*Streptococcus*	0.31%	17.68%^***^	<0.001
*Escherichia-Shigella*	5.89%	1.41%^**^	<0.01
*Eubacterium_hallii_group*	3.22%	6.45%^*^	<0.05
*Enterococcus*	0.02%	3.68%^**^	<0.01
*Agathobacter*	5.41%	1.57%^***^	<0.001
*Klebsiella*	5.58%	1.36%^*^	<0.05

*P < 0.05, **P < 0.01, ***P < 0.001.

At the genus level, NERD patients exhibited increased abundances of *Blautia*, *Bifidobacterium*, *Streptococcus*, the *Eubacterium_hallii_group*, and *Enterococcus* (all *p* < 0.05), while *Faecalibacterium*, *Bacteroides*, *Escherichia-Shigella*, *Agathobacter*, and *Klebsiella* were significantly decreased (all *p* < 0.05; Figure 4, Table 1).

#### Gut microbial biomarkers for discriminating NERD from HCs

**Figure 5:**
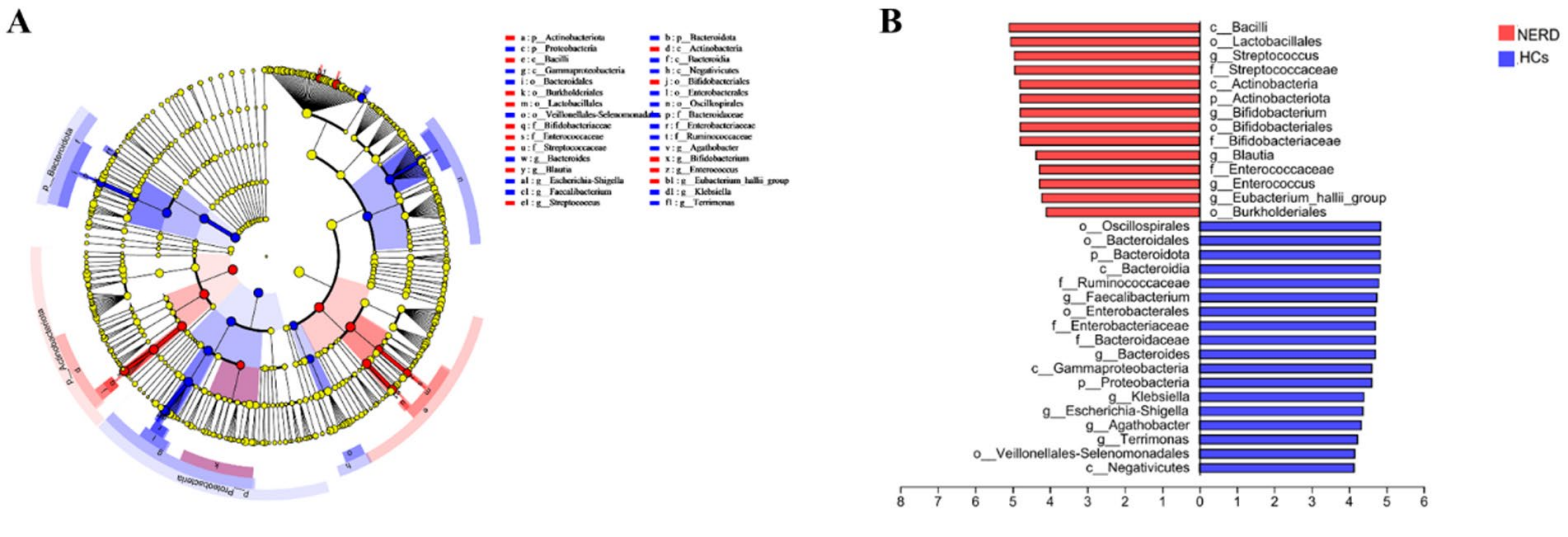
Linear discriminant analysis effect size (LEfSe) analysis of gut microbiota composition between NERD and HC groups. Only taxa with an LDA score >4 and *p* < 0.05 are shown. **(A)** Histogram of LDA scores indicating differentially abundant taxa between groups. **(B)** Cladogram representing taxonomic relationships among differentially abundant taxa. Red indicates taxa enriched in NERD patients; green indicates taxa enriched in HCs.

**Figure 6:**
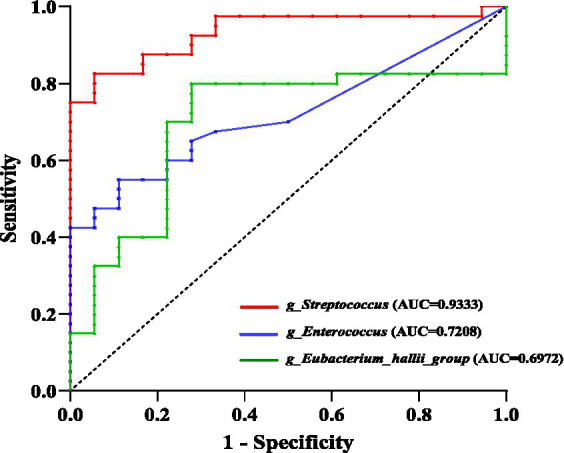
Receiver operating characteristic (ROC) curve of *Streptococcus* for distinguishing NERD patients from HCs.

LEfSe analysis identified 32 taxa that significantly differed between NERD and HC groups [linear discriminant analysis (LDA) value >4, *p* < 0.05]. Taxa enriched in HCs included Bacteroidota, Bacteroidia, Negativicutes, Bacteroidales, Oscillospirales, Enterobacterales, Veillonellales-Selenomonadales, Ruminococcaceae, Enterobacteriaceae, Bacteroidaceae, *Faecalibacterium, Bacteroides, Escherichia-Shigella, and Agathobacter*. whereas Bacilli, Lactobacillales, Streptococcaceae, Enterococcaceae, *Streptococcus*, *Enterococcus*, and *Eubacterium_hallii_group* were markedly enriched in the NERD group (Figure 5). The receiver operating characteristic (ROC) curve analysis further showed that *Streptococcus* could distinguish NERD patients from HCs with an area under the curve (AUC) value of 0.9333 (*p* < 0.0001; Figure 6).

## Discussion

NERD is the most common subtype of GERD, yet its underlying pathophysiological mechanisms remain incompletely understood. In this study, we systematically characterized the gut microbiota profile in patients with NERD using 16S rRNA gene sequencing. Our results revealed significant gut microbial dysbiosis in NERD patients, marked by reduced alpha diversity and altered community structure.

The dynamic balance of the gut microecology plays an irreplaceable role in human health. Healthy adults have a rich variety of intestinal microbiota species with high diversity, while PPIs could shift the gut microbiota toward an unhealthy state. A key finding of this study was the significantly lower alpha diversity (richness and evenness) in NERD patients compared to HCs, consistent with previous reports linking reduced microbial richness to gastrointestinal disorders (Jackson et al., 2016). In contrast, a study (Yang et al., 2021) reported that the species complexity of the gut microbiota in NERD patients was significantly higher than that in healthy individuals. This discrepancy may be attributed to differences in the use of acid suppressants and the duration of their administration. Beta diversity analysis further confirmed distinct clustering between NERD and HCs, supporting the notion that gut microbiota composition is notably altered in NERD. This reduction in diversity may reflect a less resilient and functionally imbalanced gut ecosystem, potentially contributing to symptom persistence and disease chronicity.

At the phylum level, we observed a significant decrease in Bacteroidota and Proteobacteria, alongside an increase in Actinobacteriota in NERD patients. Previous research has confirmed that, compared to healthy individuals, patients with chronic constipation show a significant increase in the abundance of Actinobacteriota and a significant decrease in the abundance of Bacteroidetes, suggesting that these microbial changes may be secondary to alterations in intestinal motility (Ding et al., 2022; Xie et al., 2018). It indicated that dysbiosis in upper gastrointestinal motility disorders often involves a reduction in beneficial phyla and an increase of opportunistic taxa. Notably, the decrease in Bacteroidota, which includes many commensals involved in polysaccharide fermentation and immune regulation, may impair intestinal barrier function and promote low-grade inflammation—a potential mechanism linking gut microbiota to NERD pathogenesis (Herfindal et al., 2022; Satokari, 2020).

The genera *Faecalibacterium*, *Bacteroides*, and *Streptococcus* were served as key microbial markers distinguishing NERD from HCs. The results of this study indicated that the relative abundances of *Faecalibacterium* and *Bacteroides* in the gut microbiota of NERD patients are significantly lower compared to HCs. *Faecalibacterium* was abundant in the fecal microbiome of HCs and served as one of the key beneficial bacteria by producing butyrate to maintain intestinal health. Its increased abundance was positively correlated with the alleviation of intestinal symptoms (Faden, 2022; Ponziani et al., 2020). Research by Valles-Colomer et al. (2019) demonstrated that *Faecalibacterium* was positively associated with higher quality-of-life indicators. *Bacteroides* was generally considered a commensal bacterium in the gut microbiota, which could inhibit the colonization of potential pathogens in the gastrointestinal tract and played a role in preventing *Clostridioides* difficile infection. It enhanced intestinal homeostasis and regulated gut function through the secretion of immunomodulatory factors (Hiippala et al., 2018; Hopkins and Macfarlane, 2002). The genus *Blautia* also exhibited a relatively high abundance in NERD. Systematic review evidence indicates an elevated abundance of *Blautia* in patients with depression (Barandouzi et al., 2020), though its specific role in human health remains to be determined (Durand et al., 2017). The relative abundance of *Enterococcus* was higher in NERD patients. To some extent, the conclusion that gut microbiota was associated with quality of life in NERD. Previous studies have shown that the use of PPIs could significantly increase the abundance of potentially pathogenic species, such as *Enterococcus*, *Streptococcus*, *Staphylococcus*, and *Escherichia coli* (Imhann et al., 2016), which was consistent with the findings of this study. Therefore, regulating the imbalanced gut microbiota may be beneficial for improving the quality of life in NERD patients.

The LEfSe in this study revealed that the class Bacilli and order Lactobacillales were significantly enriched in NERD patients. A previous study had reported that the pathogenic Bacilli posed a significant threat to human health, dominating various infectious diseases including gastroenteritis (Mallozzi et al., 2010). *Bifidobacteria* and *Lactobacilli* were widely recognized as common beneficial bacteria. A study has shown that the abundance of *Bifidobacterium* and *Lactobacillus* in the intestinal microbiota of GERD patients significantly decreased after 8 and 12 weeks of PPIs use (Li and Hu, 2019). Li et al. (2011) also observed that long-term PPIs administration led to a marked reduction in fecal *Bifidobacterium* and *Lactobacillus* levels, which contradicted the findings of our study. However, the research by Hojo et al. (2018) noted an increase in *Lactobacillus* with PPIs use, consistent with our results. It remained unclear whether the PPIs-associated changes in the abundance of these bacteria exerted harmful or beneficial effects on human health. The research findings on the effects of PPIs use in clinical practice on intestinal beneficial bacteria showed discrepancies. Considering that the composition of gut microbiota is dynamic and susceptible to various factors such as age, gender, geography, and dietary habits, this was one of the reasons for the inconsistent results. Additionally, an increase in the number of *Lactobacillus* was also observed in disease states such as type 2 diabetes mellitus and Parkinson’s disease (Hasegawa et al., 2015; Sato et al., 2014). Cannon et al. (2005) reported hundreds of cases of *Lactobacillus*-related infections, suggesting that *Lactobacillus*, as a probiotic, was defined differently under various circumstances. *Bacteria* beneficial to healthy individuals could become opportunistic pathogens, causing severe impacts on hosts with gut microbiota dysbiosis. Other studies have reported a significant increase in the abundance of *Bifidobacterium* and *Lactobacillus* in the feces of patients with active ulcerative colitis, suggesting that probiotic supplementation should be more cautiously considered during the active phase of inflammatory bowel disease (Wang et al., 2014; Song et al., 2015). This indicated that probiotic supplementation to regulate gut microbiota dysbiosis was not suitable for all disease conditions. Overall, at the genus level, the composition of intestinal microbiota in NERD patients was complex, with disrupted distribution of beneficial and harmful bacteria, warranting further in-depth research on its impact on human health.

The relative abundances of Streptococcaceae, Enterococcaceae, *Streptococcus*, and *Enterococcus* were significantly increased in NERD patients, serving as important microbial markers distinguishing them from HCs. *Streptococcus* is a common type of Gram-positive coccus, with studies showing higher abundance in the gut microbiota of gastric cancer patients compared to healthy individuals (Zheng et al., 2019). *Streptococcus* is a common commensal organism in the human oral cavity, throat, and nasal passages. Gastric acid normally acts as a barrier to inactivate ingested microorganisms and prevent bacterial migration from upper regions like the mouth to the lower digestive tract (Howden and Hunt, 1987). PPIs inhibit gastric acid secretion, weakening the gastric acid barrier that normally inactivates ingested microorganisms. This barrier dysfunction leads to the colonization and enrichment of *Streptococcus* in the gut, which may partially explain the observed increase in *Streptococcus* among NERD patients in this study. A meta-analysis revealed that the use of PPIs increased the abundance of species such as Enterobacteriaceae, Lactobacillaceae, Streptococcaceae, and Actinomycetaceae, while decreasing the abundance of Bifidobacteriaceae and Ruminococcaceae (Macke et al., 2020), which showed both similarities and differences with the findings of this study. Ruminococcaceae are a group of strict anaerobic bacteria that play a crucial role in maintaining gut health through their production of butyrate and short-chain fatty acids. A reduction in their abundance has been associated with inflammatory bowel disease (Morgan et al., 2012). A systematic review indicated that patients with depression exhibited high abundances of Actinobacteria, Bifidobacteriaceae, Streptococcaceae, *Streptococcus*, *Blautia*, and *Klebsiella* in gut microbiota (Barandouzi et al., 2020), which was consistent with the findings of this study showing significant enrichment of Actinobacteria, Bifidobacteriaceae, Streptococcaceae, *Streptococcus*, and *Blautia* in NERD patients. This suggested that NERD patients had a relatively higher abundance of gut microbes associated with negative psychological states, and the gut microbiota may serve as the material basis for the psychosomatic comorbidity in NERD patients. Moreover, ROC curve analysis further revealed that *Streptococcus* exhibited high accuracy in distinguishing NERD patients from HCs, serving as a key microbial biomarker characteristic of NERD disease. *Streptococcus* could be used as novel targets for clinical non-invasive diagnostic biomarkers and therapeutic interventions for NERD in the future. The elevated levels of *Lactobacillus* and *Bifidobacterium* may be associated with bacterial translocation or increased harmful bacterial infections induced by PPIs use (Huang et al., 2022). Overall, NERD patients demonstrated complex gut microbiota composition with disrupted species structure, indicating an unhealthy intestinal state.

Our study has several limitations. First, we adopted a cross-sectional case–control design, which can effectively identify differences in gut microbiota between NERD patients and HCs. It remains unclear whether the observed dysbiosis contributes to NERD pathogenesis or results from the disease itself and/or its treatment with PPIs. While 16S rRNA sequencing provides limited resolution at the species level and functional inference. Second, although we excluded the factors most likely to interfere with the microbiota (probiotics and antibiotics), the 2-week antibiotic washout period in this study was relatively short, which may have a potential impact on microbial composition results. Future studies should adopt longitudinal designs (e.g., sampling before and after PPIs treatment) and randomized controlled trials, combined with animal experiments, employing metagenomic sequencing, to verify the causal relationship between specific gut microbiota and NERD, and explore whether regulating target bacteria can alleviate NERD symptoms.

## Conclusion

In this study, we characterized and identified the composition and diversity of fecal microbiota in NERD patients and compared them to healthy individuals using 16S rRNA gene sequencing. Alterations of gut microbial composition and structure were observed at different taxonomic levels in NERD patients, suggesting the critical role of gut microbiota in the pathogenesis and progression of NERD. Notably, the enrichment of *Streptococcus* and depletion of Bacteroidota and *Faecalibacterium* may serve as potential microbial biomarkers for NERD diagnosis. Thus, comprehensive characterization of the NERD-associated fecal microbiota will lay the foundation for clinical diagnostic research and identification of potential therapeutics in the future.

## Data Availability

The data presented in this study are publicly available in the NCBI (https://www.ncbi.nlm.nih.gov/), accession PRJNA1451217.

## Footnotes

1
https://github.com/OpenGene/fastp

2
https://ccb.jhu.edu/software/FLASH/index.shtml

3
http://drive5.com/uparse/

4
https://sourceforge.net/projects/rdp-classifier/

5
http://huttenhower.sph.harvard.edu/galaxy/root?tool_id=lefse_upload
